# Prevalence and risk factors of hepatitis C virus infection in Armenia, 2021

**DOI:** 10.1093/eurpub/ckac131.392

**Published:** 2022-10-25

**Authors:** A Demirchyan, S Sahakyan, L Aslanyan, L Mushegyan, D Muradyan, A Mozalevskis, N Sargsyants, G Ghukasyan, V Petrosyan

**Affiliations:** 1 Turpanjian College of Health Sciences, American University of Armenia, Yerevan, Armenia; 2 WHO Regional Office for Europe, Copenhagen, Denmark; 3 National Centre for Infectious Diseases, Yerevan, Armenia; 4 WHO Country Office in Armenia, Yerevan, Armenia

## Abstract

**Background:**

Liver damage caused by hepatitis C virus (HCV) is common, especially in low- and middle-income countries. Chronic HCV infection is among the leading causes of chronic liver disease, cirrhosis and hepatocellular carcinoma. Data on prevalence and risk factors of HCV infection are important for planning effective interventions to fight the virus. This study investigated the prevalence of HCV, its genotypes and factors associated with chronic HCV infection in Armenia.

**Methods:**

The study included 3838 individuals 18 years and older selected via stratified two-stage cluster sampling from all regions of Armenia. Anti-HCV antibodies were detected using a third generation immunoassay. Those testing positive were further tested by Polymerase Chain Reaction and genotyping. Shortly after testing, the participants underwent a telephone survey. Logistic regression model was fitted to identify factors associated with chronic HCV infection.

**Results:**

The participants mean age was 49.5 years, 70.0% were female. The prevalence of HCV antibodies weighted by age and sex was 1.9% (95% CI 1.5, 2.3), and chronic HCV infection - 0.7% (95% CI 0.4, 0.9), with genotype 3 being the most common (41.7%), followed by genotypes 2 (37.5%) and 1 (20.8%). The prevalence of both antibodies and chronic infection were higher among 50-69 years old (3.4% and 1.3%, respectively). In weighted analysis, the risk factors for chronic HCV infection included male sex (95% CI 1.23, 11.59), having tattoos (95% CI 1.10, 7.80), and reporting liver disease (95% CI 1.24, 14.61). Being employed was protective (95% CI 0.14, 0.93).

**Conclusions:**

This study was the first attempt to measure the prevalence of HCV infection among the general population of Armenia, creating prerequisites for estimating the HCV-related disease burden and developing strategies to cope with it. The identified risk factors demonstrate that there is still room for strengthening safety measures to prevent the transmission of HCV in Armenia.

**Key messages:**

• The prevalence of HCV antibodies is 1.9% among adult population of Armenia, increasing with age. Over one-third of seropositive cases have chronic infection caused by HCV genotypes 3, 2 or 1.

• Having tattoos is associated with higher risk of being infected with HCV, demonstrating the need for strengthening safety measures during similar procedures to prevent viral transmission.

